# Mitochondrial toxicants in Xian-Ling-Gu-Bao induce liver injury by regulating the PI3K/mTOR signaling pathway: an in vitro study

**DOI:** 10.1186/s12906-022-03798-5

**Published:** 2022-12-01

**Authors:** Shujuan Piao, Hongwei Lin, Xia Tao, Wansheng Chen

**Affiliations:** 1 Department of Pharmacy, Second Affiliated Hospital of Naval Medical University, Shanghai, 200003 China; 2 Traditional Chinese Medicine Resource and Technology Center, Shanghai University of Traditional Chinese Medicine, Shanghai, 201203 China

**Keywords:** Drug-induced liver injury, Xian-Ling-Gu-Bao, Mitochondrial dysfunction, Bioenergetics, Apoptosis, Autophagy

## Abstract

**Background:**

Drug-induced mitochondrial toxicity is thought to be a common mechanism of drug hepatotoxicity. Xian-Ling-Gu-Bao (XLGB) oral preparation is a commonly used drug for osteoporosis in China. Classical safety evaluation studies have shown that the entire preparation and six Chinese herbal medicines have high safety, but the incidence of drug-induced liver damage due to XLGB remains high, the mechanism and toxic substances causing liver injury are still unclear. The purpose of this study is to identify compounds with potential mitochondrial liabilities in XLGB, and to clarify their underlying mechanisms and related pathways.

**Methods:**

The mitochondrial function analysis was performed using an extracellular flux assay, which simultaneously monitored both oxygen consumption rate (OCR) and extracellular acidification rate (ECAR). Through network pharmacology and in vitro experimental verification, the potential protein targets, signaling pathways and molecular mechanism of mitochondrial toxicity have been studied.

**Results:**

We observed a significant decrease in mitochondrial respiration of Psoraleae Fructus and its five compounds in fundamental bioenergetics parameters such as basal respiration, ATP-linked production and maximal respiration, indicating mitochondrial dysfunction. The network pharmacology results showed that the influence of XLGB on mitochondrial dysfunction was closely related to PI3K-Akt signaling pathway, mTOR signaling pathway and Apoptosis. Western blot showed that the levels of mTOR, p-mTOR (Ser2448), Raptor, PI3K (p110α), Beclin 1, ATG5 and Caspase-9 were up-regulated after treatment with psoralidin, psoralen and bavachin, and the expression of Bcl-2 was down-regulated after bavachinin treatment.

**Conclusions:**

The hepatotoxicity of XLGB is associated with mitochondrial dysfunction. Five compounds in Psoraleae Fructus showed mitochondrial damage, they are psoralidin, isobavachalcone, bavachinin, bavachin and psoralen, especially psoralidin showed significant reduction in reserve capacity and respiratory control ratios. The molecular mechanism is related to the activation of PI3K/mTOR signaling pathway to inhibit autophagy and induce mitochondrial apoptosis.

**Supplementary Information:**

The online version contains supplementary material available at 10.1186/s12906-022-03798-5.

## Background

Drug-induced liver injury (DILI) is a typical adverse reaction in clinical practice, and it is also the main reason for the withdrawal of drugs from the market [1]. Most DILI cases are idiosyncratic and unpredictable. Idiosyncratic DILI (iDILI) is believed to be influenced by multiple factors, including immune system problems, infections and genetic factors, and the underlying mechanisms remain unclear. However, this doesn’t mean that some drugs that cause iDILI have no early warning in preclinical studies. In fact, as screening techniques continue to improve, those drugs that cause iDILI have shown damage to hepatocytes, especially to mitochondria [2]. Many of the drugs that have been withdrawn from the market or received FDA Black Box warnings have shown or have the potential to impair mitochondrial function [3]. During DILI, mitochondria may be as target of drugs causing idiosynctatic DILI [4]. Many idiosyncratic toxicants can directly damage mitochondria to stress liver cells. A screening of 300 idiosyncratic toxicants that damaged mitochondrial function in liver cells found that 50–60% of the drugs caused mitochondrial dysfunction [5]. Therefore, the detection of mitochondrial toxicity to predict DILI can reduce the rate of attrition during drug development.

Generally, herbal medicines are considered safe because they occur naturally and have been used for thousands of years in therapeutic applications. But in fact, it is now clear that many herbal medicines are hepatotoxic [6]. Compared to the past, DILI caused by herbal medicines (HILI, herbal-induced liver injury) is still occurring [7, 8]. Among 7511 cases of DILI, 1874 cases were caused by herbal medicines (25.0% of DILI) [9]. China is one of the most populous countries using both standard treatment and traditional Chinese medicine (TCM). In China, the diagnosis of TCM hepatotoxicity has been widely observed from preclinical to clinical studies. However, there are still many difficulties in HILI research, because herbal medicines contain many substances, especially Chinese herbal compound preparation. It’s hard to detect the toxic substances it contains, and it’s even harder to know which ingredients cause liver damage [10].

Xian-Ling-Gu-Bao (XLGB) capsule is an over-the-counter (OTC) drug officially approved by China Food and Drug Administration (CFDA; China, Z20025337) for the treatment of osteoporosis [11]. As a traditional Chinese medicine compound preparation, XLGB is composed of six herbal medicines with the following weight percentages: Epimedii Folium (70%), Dipsaci Radix (10%), Psoraleae Fructus (5%), Salviae Miltiorrhizae Radix et Rhizoma (5%), Rehmanniae Radix(5%) and Anemarrhenae Rhizoma (5%). See Table 1 for the full scientific species names of all ingredients of XLGB. Preclinical studies in ovariectomized (OVX) rats [12, 13] and clinical trials [14, 15] demonstrated the drug’s clinical efficacy. However, from January 1, 2004 to July 21, 2016, the National Adverse Drug Reaction Monitoring Database received a total of 2665 reports about XLGB oral preparations, of which 81 cases (3.1%) were severe reports, mainly manifested as increased liver enzyme levels, increased bilirubin levels, liver cell damage, etc. The proportion of hepatobiliary system damage was significantly higher than the corresponding proportion in the overall reports. The CFDA issued a notification on the risk of liver injury caused by XLGB on December 8, 2016. But, the toxicology study information available on XLGB is limited. Safe doses of XLGB up to 1800 mg/kg (6 times the clinical dose (3 g/day)) have been reported In Sprague-Dawley (SD) rats and OVX rats [12, 16]. Wu et al. failed to observe liver damage after 90 days of treatment with 5000 mg/kg XLGB in 12-month old female rats, but they found that XLGB could induce inflammatory stress rat liver injury [17]. Until now, whether XLGB hepatotoxicity is related to iDILI, and what mechanism and toxic substances induce liver damage are still unknown. Therefore, looking for the mitochondrial targeted herbs and ingredients that cause XLGB liver toxicity may be the first step to help reveal the mechanism of liver damage.

**Table 1:** Full scientific species names for all ingredients of XLGB

No.	Plant name in English	Scientific name
1	Epimedii Folium	*Epimedium brevicornu* Maxim.
2	Dipsaci Radix	*Dipsacus asper* Wall. ex Henry
3	Psoraleae Fructus	*Psoralea corylifolia* L.
4	Salviae Miltiorrhizae Radix et Rhizoma	*Salvia miltiorrhiza* Bge.
5	Rehmanniae Radix	*Rehmannia glutinosa* Libosch.
6	Anemarrhenae Rhizoma	*Anemarrhena asphodeloides* Bge.

Effects of mitochondrial toxicants on mitochondrial function can be assessed by measuring oxygen consumption rates (OCR). Previously, the measurement of OCR was usually performed using Clark oxygen electrodes, but these electrodes were not capable of medium throughput screening. The recently developed multiwall, plate-based Seahorse XF96 Extracellular Flux (XF) analyzer addresses the need for a flexible and higher throughput OCR assay technology [18]. Equipped with a solid-state fluorescent sensor to measure OCR and a second sensor that can be used to measure proton concentration, the Seahorse analyzer provides extracellular acidification rate (ECAR). Thus, full picture of the energy metabolism curve can be well assessed using the Seahorse XF analyzer.

Due to the complex composition and multi-target characteristics of TCM, network pharmacology has shown great advantages in the study of the mechanism of TCM by revealing the relationship between drugs, targets and diseases [19]. This study aimed to identify compounds in XLGB with potential mitochondrial liabilities, predict and analyze the related targets of toxic components through network pharmacology, and further explore their mechanism of action in the occurrence of liver injury.

## Materials and methods

### Materials

The human hepatoblastoma cell line HepG2 was obtained from Chinese Academy of Sciences Cell Bank (Shanghai, China). Culture media and agents were purchased from Gibco (Waltham, USA). CellTiter-Glo® 2.0 cell viability assay kit was purchased from Promega (Madison, USA). Seahorse consumables were all purchased from Seahorse Biosciences (North Billerica, USA). The pure test compounds were purchased from Dalian Meilun Biotechnology Co., Ltd. (Dalian, China). Xian-Ling-Gu-Bao capsule was produced by Tongjitang Pharm Co., Ltd. (Guiyang, China), and the six TCMs in it were purchased from Tongling Hetian Herbal Pieces Co. Ltd. (Anhui, China). Acetonitrile and formic acid for HPLC were purchased from Sigma-Aldrich (St. Louis, USA). The methanol and ethyl acetate used in extracting the samples were all analytical grade.

### Determination of the main compounds in Psoraleae Fructus

#### Sample preparation

1 g of XLGB powder was added into a 50 mL volumetric flask, added with 75% methanol, ultrasonicated (SY-800, 40KHZ, Shanghai Weimi Technology Co., Ltd., Shanghai, China) for 30 min, and then made to volume at room temperature. Then accurately measured 3 mL of the solution into a 10 mL volumetric flask, and added methanol to make up.

A 10 g fine-grinded powder sample of Psoraleae Fructus was dissolved in 100 mL of water, and extracted with ethyl acetate for three times, each 100 mL, to obtain 0.39 g of the extract, which was then diluted with methanol into a 5 mL volumetric flask.

#### Fingerprint analysis of Psoraleae Fructus and XLGB

The Agilent 1200 series HPLC system (Agilent Technologies, USA) was used for the chromatographic fingerprint analysis. The equipment was equipped with a quatemary solvent delivery pump (G-1311C), an online degasser (G-1322A), a diode array detector (G-4212B DAD), a column temperature controller (G-1316A) and Agilent ChemStation. The column used was an Agilent Eclipse plus C_18_ column (4.6 mm × 250 mm, 5 μm), the flow rate was set to 0.8 mL/min, the sample volume was 10 μL, and the detection was performed at 270 nm and 35 °C. Acetonitrile (A)-0.1% aqueous formic acid (B) was used as the mobile phase for separation, and the elution gradient of the mobile phase was as follows (A%): 0–5 min, 2% → 2%; 5–80 min, 2% → 36%; 80–95 min, 36% → 48%; 95–110 min, 48% → 80%; 110–112 min, 80% → 100%.

#### HPLC-TOF-MS analysis of Psoraleae Fructus

Mass spectra in positive and negative ion mode were acquired on an Agilent 6538 UHD and accurate mass Q-TOF/MS. After separation on HPLC system (Agilent 1290 Infinity UPLC, Agilent Technologies, USA), mass spectra were achieved on Agilent 6538 UHD and Accurate-Mass Q-TOF/MS (Agilent Technologies, USA) with the following conditions: electrospray ion source (Dual ESI+); capillary pressure, 3500 V; atomized gas pressure, 45 psi; dry gas temperature, 350 °C; debris voltage, 120 V; data acquisition range, m/z100–1100. Before the experiment, TOF/MS automatically corrected the mass coordinate axis by real-time injection of reference solution (Agilent Technologies, USA), and the flow rate of reference solution was 100 μL/min; Negative ion mode detection: electrospray ion source (Dual ESI); other conditions are the same as positive ion mode.

### Cell culture

HepG2 Cells were cultured in DMEM (L-Glutamine 4.00 mM, Glucose 4.50 mg/L, and Sodium Pyruvate 110 mg/L) added with 10% FBS, 100 U/mL penicillin and 100 μg/mL streptomycin, and then maintained in six-pore suspension culture plates at concentration of 10,000 cell/mL with 5% CO_2_ at 37 °C.

### Cell viability assay

Determination of cell viability was by the Cell Titer−Glo® (CTG) 2.0 Assay (Promega, Madison, USA). 5000 cells per well were seeded in 96-well plates and adhered to the plates overnight. Cells were disposed with test compounds diluted at 0.002, 0.02, 0.2, 2, 20, and 200 μg/mL final assay concentrations in serum-free medium, and an equal amount of Cell Titer Glo reagent was supplemented 72 hours later. Transfer half the total volume of each well to 96-well opaque plates, and the emitted light was measured by Multi-Plate Reader (Biotek Synergy 2) after ten minutes.

### Measurement of cell bioenergetics

#### Extraction of herbal medicines

200 g of herbal powder was added to 2000 mL of water, extracted with rotary vacuum evaporator (N-1001, Shanghai EYELA Technology Co., Ltd., Shanghai, China) 3 times, 2 hours each time, combined with the extract, concentrated and dried (Vacuum drying oven, 9715, Changshu Pharmaceutical Marchinery Factory Co., Ltd., Jiangsu, China).

#### Seahorse assay

The bioenergetic function of HepG2 cells was measured by Agilent Seahorse XFe96 Analyzer (Seahorse Biosciences, USA). Day prior to assay, HepG2 cells were inoculated into the cell culture plates of Seahorse analyzer at the density of 20,000 cells per well, adhered and grew in 37 °C humidified incubator (containing 5% CO_2_), and hydrated the XF extracellular flux sensory cartridge. On the experimental day, the appropriate concentrations of herbal extracts and compounds were added and continued to incubate for 2 hours to make the cells reach about 80% confluency. Then, the cell plate medium was replaced with XF Base DMEM Medium (pH 7.4) containing 1.0 M glucose, 100 mM sodium pyruvate and 200 mM of glutamine solution, and incubated at 37 °C without CO_2_ for half an hour. Finally, Oligomycin (1.5 μM), FCCP (1 μM) and the mixture of Rotenone and Antimycin A (0.5 μM) were injected sequentially according to the instrument setting procedure, and then the data analysis was carried out [20].

**Fig. 1:**
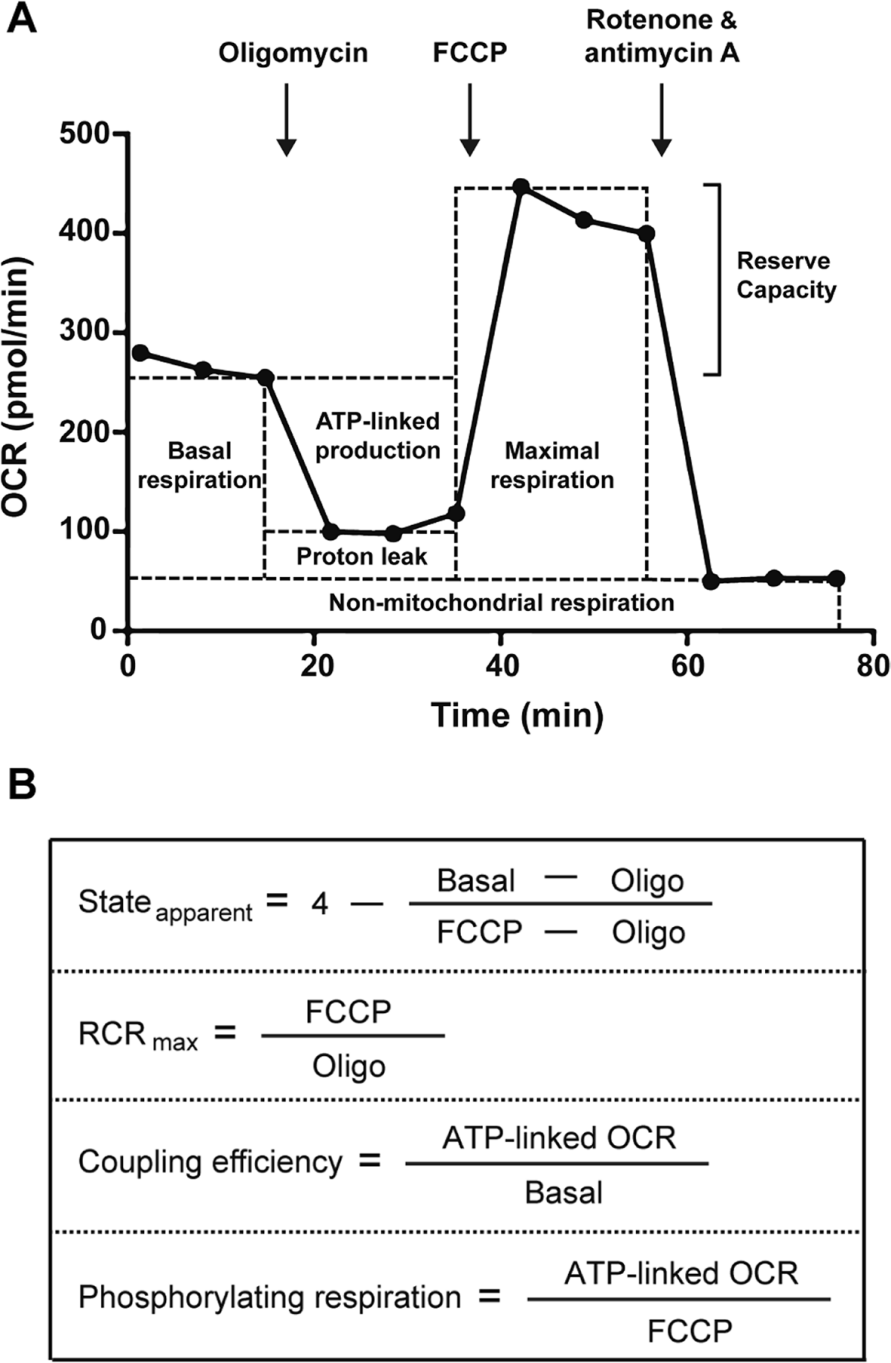
Overview of different respiratory parameters obtained by Seahorse assay. (A) This example is from control HepG2 cells with sequential additions of mitochondrial stressors Oligomycin, FCCP, and the mixture of Rotenone and Antimycin A, allowing evaluation of the mitochondrial function parameters: basal respiration, ATP-linked production, proton leak, maximal respiratory capacity, reserve capacity, and non-mitochondrial respiration. (B) Using the mitochondrial function parameters described above, the respiratory flux control ratios can be derived. In these equations, Basal stands for the basal respiration, Oligo stands for oligomycin-insensitive OCR, which is the proton leak OCR, and FCCP stands for FCCP-stimulated OCR, which is the maximal respiration OCR

The bioenergetic profile generated by the sequential addition of mitochondrial inhibitors can delineate the details of the respiratory chain (Fig. 1 a). From the bioenergetic profile, we can derive six parameters about mitochondrial function, which are basal respiration, ATP-linked production, proton leak, reserve capacity, maximal respiratory capacity and non-mitochondrial respiration [21]. Applying these mitochondrial function parameters allows the derivation of respiratory flux control ratios: State_apparent_, respiration control ratio (RCR), coupling efficiency and phosphorylating respiration (Fig. 1b). The classical State 3 and State 4 observed in isolated mitochondria cannot be replicated in intact cells, whereas an intermediate transition state is proposed in intact cells, termed State 3.5 (State_apparent_). The cells apparent respiratory state can be determined using State_apparent_ [22]. Based on the assumption that State 3 respiration equals the rate determined after the addition of FCCP and State 4 equals the rate after the addition of oligomycin, the equation of State_apparent_ and RCR_max_ can be obtained [23]. Coupling efficiency is calculated as the section of basal mitochondrial respiration used for ATP synthesis, and phosphorylating respiration is expressed as the fraction of respiration used to generate ATP under conventional conditions [22, 24].

### Network pharmacology analysis

The canonical SMILES of the compounds were collected in the PubChem database (http://pubchem.ncbi.nlm.nih.gov) and then used in Swiss Target Prediction (http://www.swisstargetprediction.ch) to predict the molecular targets of the compounds. The UniProt database (https://www.uniprot.org) and Comparative Toxicogenomics Database (http://ctdbase.org/) further validated the targets of the compounds. Targets associated with mitochondrial toxicity were obtained using GeneCards (https://www.genecards.org) and Comparative Toxicogenomics Database with the keyword “mitochondrial dysfunction”. Uploading selected targets to the online Venn diagram (https://bioinfogp.cnb.csic.es/tools/venny/) yielded common targets for compounds and mitochondrial toxicity. Protein-protein interactions (PPIs) were identified using the STRING database (https://string-db.org). GO and KEGG pathway enrichment analysis was performed using WebGestalt (http://www.webgestalt.org). Network Visualization was conducted with Cytoscape software (version 3.7.1).

### Western blot analysis

HepG2 cells were rinsed with PBS and then lysed in lysis buffer (containing Tris-HCl 62.5 mM, DTT 100 mM, 10% glycerol, 2% SDS, and pH 6.8). The protein concentration was determined by Pierce BCA protein assay kit (Thermo Scientific, USA), and equal amounts of protein was separated by SDS-PAGE (Yamei Biotec, China) and then electrophoretically transferred to Immobilon-E PVDF Membrane (merck millipore, Germany). After blocking using 5% BSA (Thermo Scientific, USA) for 1 hour at room temperature, the transferred membranes were incubated overnight with primary antibodies (1:1000) at 4 °C. Next, the membranes were washed by TBST (Tween-20 in Tris-buffered saline), added with secondary antibodies (1:1000), and incubated for 2 hours at room temperature. Protein bands were visualized by ODYSSEY CLx (LI-COR, USA) with IR700/IR 800 labeled secondary antibody and analyzed using Image Lab software (Image Studio Lite v3.1, LI-COR, USA).

### Statistical analysis

Data were statistically analyzed using SPSS 23.0 (SPSS, USA) and Graphpad Prism® 9 software (GraphPad, USA). Statistically significant differences were evaluated with Student’s paired *t*-test, one-way Analysis of Variance (ANOVA). A *p*-value < 0.05 is statistically significant. The IC_50_ values were obtained using a nonlinear regression curve fit in Graphpad Prism.

## Results

### Herbal medicines with mitochondrial toxicity in XLGB

**Fig. 2:**
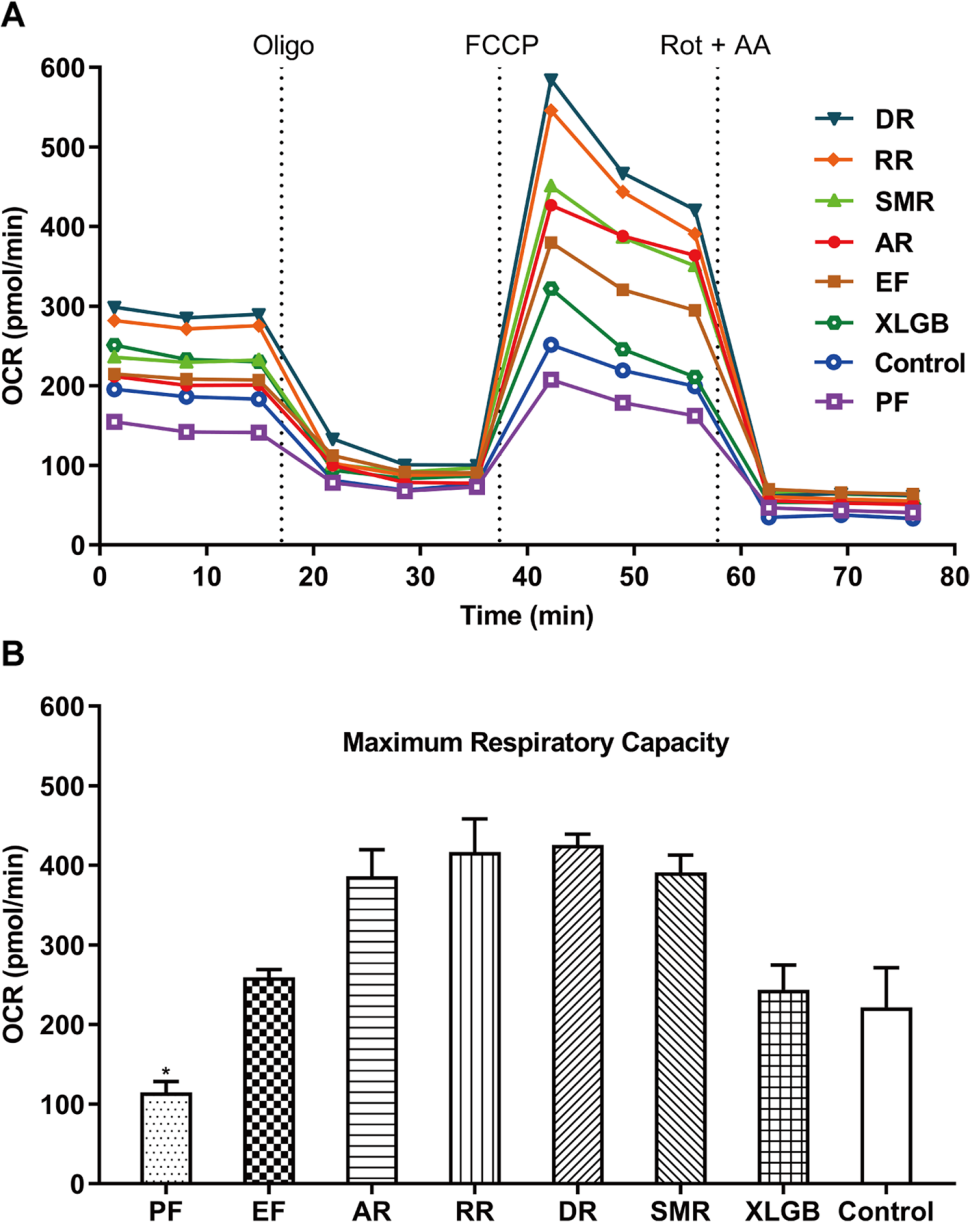
Effects of the six herbal medicines in XLGB capsule on the cellular bioenergetics of HepG2 cells. (A) Real time measurement (mean) of OCR was measured using Seahorse assay, and the concentrations of the six herbal extracts were all at 800 μg/mL. (B) From panel A, maximum respiratory capacity of the six herbal medicines linked oxygen consumption rates was calculated. Abbreviations and the corresponding plant names are as follows: DR (Dipsaci Radix), RR (Rehmanniae Radix), SMR (Salviae Miltiorrhizae Radix et Rhizoma), AR (Anemarrhenae Rhizoma), EF (Epimedii Folium), PF (Psoraleae Fructus). Bars represent mean ± SD, *n* = 3 (*P < 0.05 versus control OCR)

In our previous work, the toxicity of the six herbal medicines in XLGB was measured using the CCK-8 assay, the IC_50_ of Psoraleae Fructus and Epimedii Folium was significantly lower than other herbs. Mitochondrial function of the six herbal medicines was tested by the Seahorse assay (Fig. 2 a). The results of mitochondrial function parameters (Fig. 2 b) showed that the maximum respiratory capacity of Psoraleae Fructus was lower than that of the control, while the other five herbal medicines have no decrease in OCR, suggesting that Psoraleae Fructus is the medicine that causes mitochondrial toxicity in XLGB.

### Compounds in Psoraleae Fructus

**Fig. 3:**
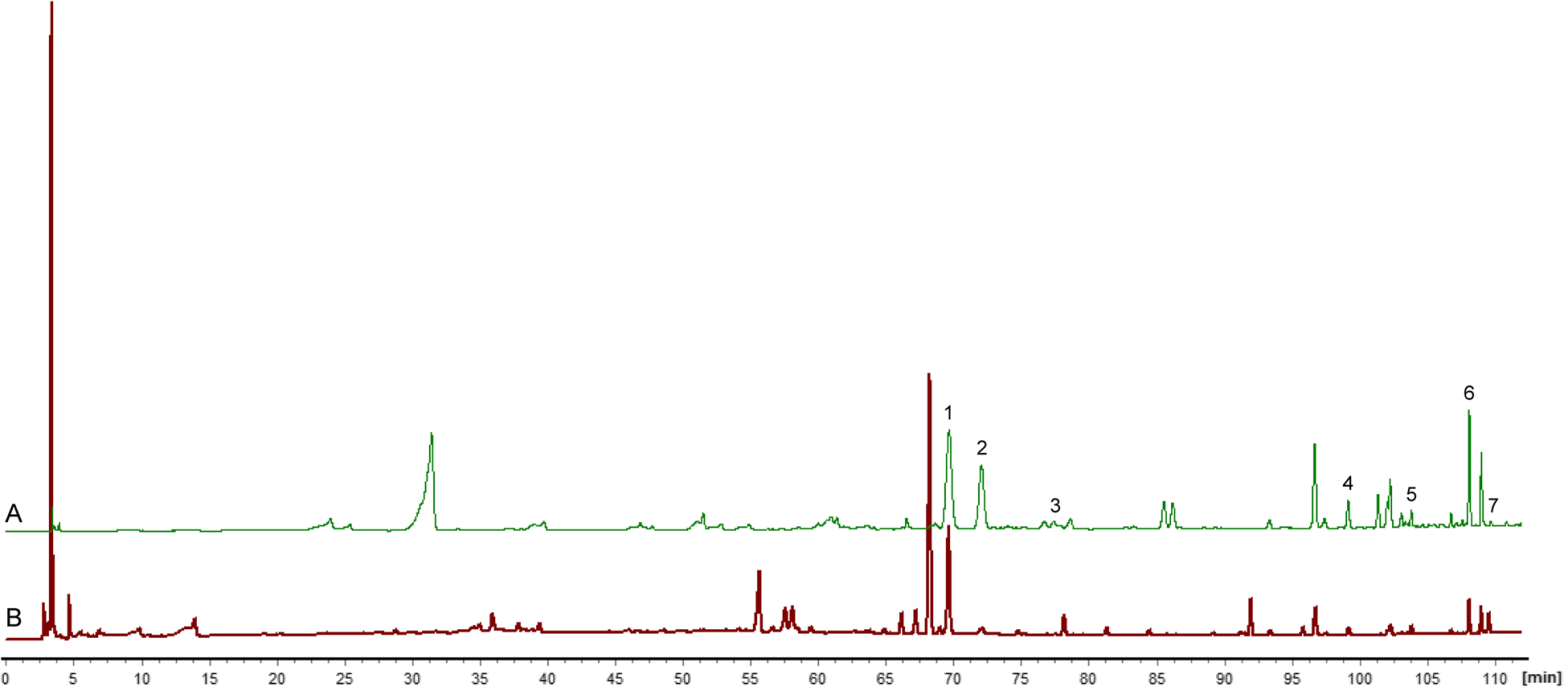
Fingerprint chromatogram of Psoraleae Fructus and XLGB. (A) Fingerprint of Psoraleae Fructus. 1. Psoralen; 2. Bavachin; 3. Bavachromanol; 4. Psoralidin; 5. Isobavachalcone; 6. Bavachinin; 7. Corylifol A. (B) Fingerprint of XLGB

Seven compounds in Psoraleae Fructus were identified by Fingerprint analysis (Fig. 3) and HPLC-TOF-MS. Several reference substances with relatively high content in ion flow diagram were selected as reference materials, the preliminary quantitative detection by HPLC-DAD method was carried out, and the content of five main components was quantitative determined, which were prepared for the subsequent determination of toxic substances (Table 2).

**Table 2:** HPLC-TOF/MS characterization of main compounds in Psoraleae Fructus

No.	Identification	Molecular weight	MS data	Contents (μg/g)
1	Psoralen	186.16	187.04 [M + H]^+^	2.63
2	Bavachin	324.14	325.04 [M + H]^+^	1.82
3	Bavachromanol	340.14	341.14 [M + H]^+^	Not available
4	Psoralidin	336.34	337.11 [M + H]^+^	0.64
5	Isobavachalcone	324.14	325.15 [M + H]^+^ 323.13 [M − H]^+^	0.51
6	Bavachinin	338.34	339.13 [M + H]^+^ 337.11 [M − H]^+^	0.98
7	Corylifol A	390.19	391.20 [M + H]^+^	Not available

### Effect of five compounds in Psoraleae Fructus on cell viability

The CTG assay determined the number of living cells in culture by quantifying the presence of ATP. The five compounds inhibited cells proliferation in a concentration- and time- dependent manner, with IC_50_s of 508.17, 26.69, 72.65, 82.34 μM for psoralidin, isobavachalcone, bavachinin and bavachin respectively, and 2.88 mM for psoralen (The figure is in Supporting Information).

### Impact of the compounds on mitochondrial function

There are two ways to define drugs as mitochondrial toxicants, direct mitochondrial toxicants and indirect mitochondrial toxicants [25]. Direct mitochondrial toxicants refer to drugs that directly lead to a decrease in basal respiratory OCR and an increase in ECAR. Drugs are classified as indirect mitochondrial toxicants if at least one of the four changes in stressor parameters below: decreased ATP-linked production OCR, maximal respiratory capacity or/and reserve capacity, or increased proton leak.

**Fig. 4:**
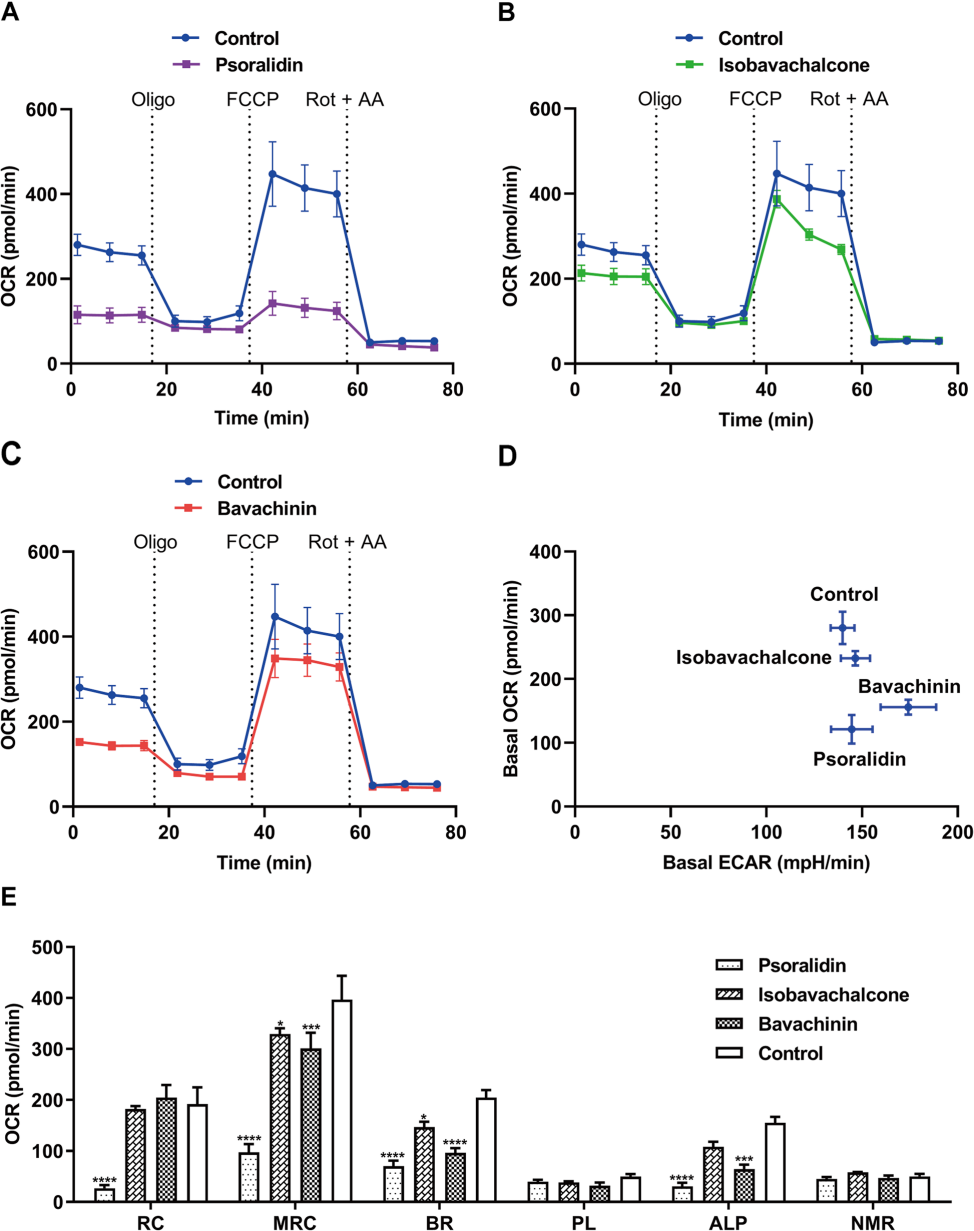
Measurement of bioenergetics profile of psoralidin, isobavachalcone, and bavachinin. OCR (A, B, and C) and ECAR (D) were measured in real time, and the concentrations of psoralidin, isobavachalcone, and bavachinin were at 446.43, 30.86, and 59.17 μM respectively. (E) Each mitochondrial parameter was calculated derived from the data in A, B and C. Abbreviations and the corresponding parameters are as follows: reserve capacity (RC), maximal respiratory capacity (MRC), basal respiration (BR), proton leak (PL), ATP-linked production (ALP), and non-mitochondrial respiration (NMR). Data represent mean ± SD, *n* = 3 (*P < 0.05, ***P < 0.001, ****P < 0.0001 versus control OCR)

**Fig. 5:**
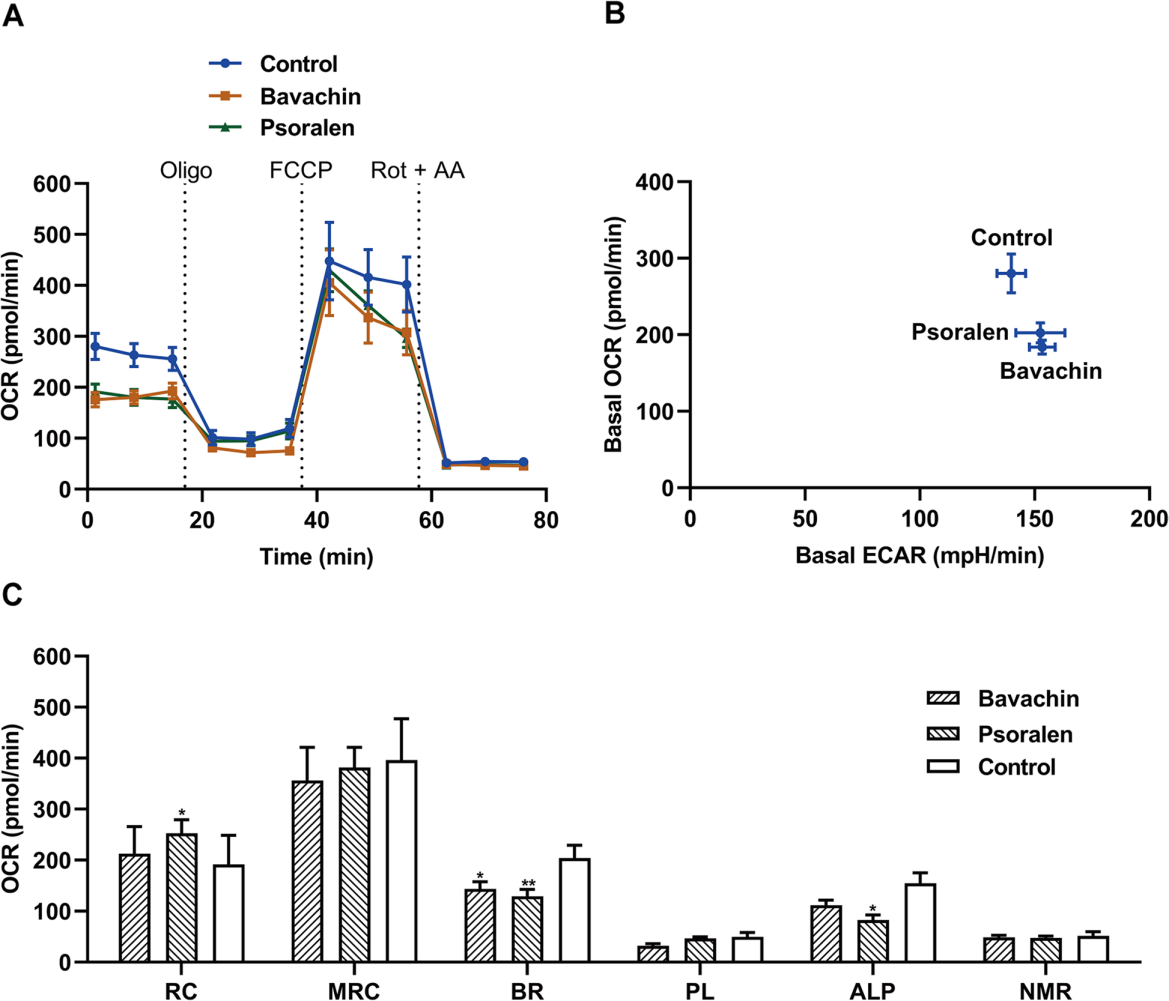
Measurement of bioenergetics profile of bavachin and psoralen. OCR (A) and ECAR (B) were measured in real time, and the concentrations of bavachin and psoralen were at 61.73 μM and 1.08 mM respectively. (C) Each mitochondrial parameter was calculated derived from the data in A. Abbreviations and the corresponding parameters are as follows: reserve capacity (RC), maximal respiratory capacity (MRC), basal respiration (BR), proton leak (PL), ATP-linked production (ALP), and non-mitochondrial respiration (NMR). Data represent mean ± SD, *n* = 3 (*P < 0.05, **P < 0.01 versus control OCR)

From the bioenergetics results of psoralidin, isobavachalcone, bavachinin, bavachin and psoralen which as the main ingredients of Psoraleae Fructus (Fig. 4 and Fig. 5), psoralidin, isobavachalcone, and bavachinin induced a decrease in basal respiration OCR (Fig. 4 a-c) and an increase in ECAR (Fig. 4 d) respectively, together with significant decreased values on OCR associated to maximal respiratory capacity and ATP-linked production (except isobavachalcone) (Fig. 4 e), indicating the three substances above are mitochondrial toxic ingredients. The decreased reserve capacity can be seen in psoralidin and isobavachalcone, but only psoralidin has a significant difference (Fig. 4 e). Bavachin and psoralen also showed decreased values on basal respiration OCR and increase valules in ECAR (Fig. 5 a-b), but only psoralen showed a significant decrease in ATP-linked production (Fig. 5 c).

**Fig. 6:**
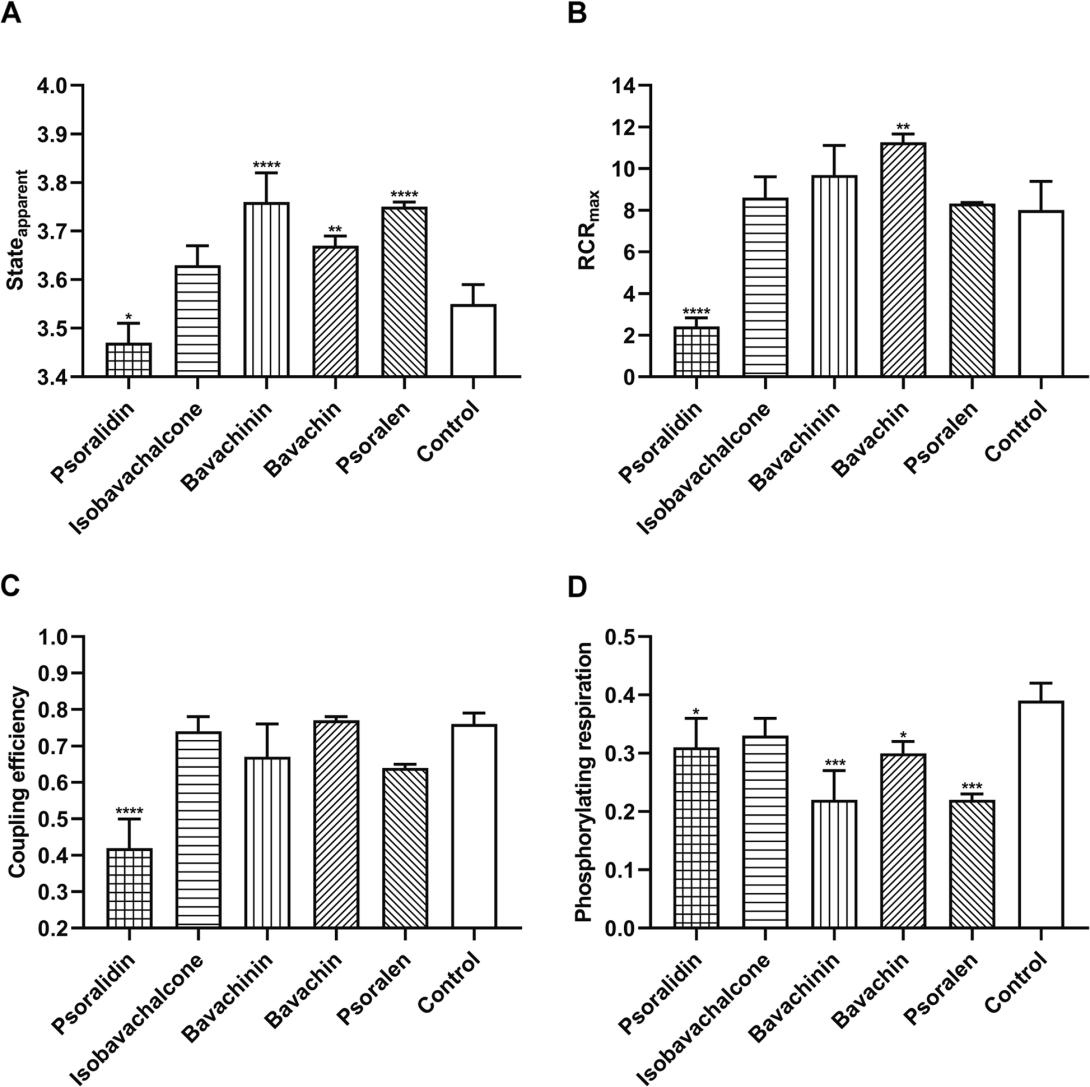
Mitochondrial respiratory flux control ratios of psoralidin, isobavachalcone, bavachinin, bavachin, and psoralen. The State_apparent_, RCR_max_, coupling efficiency and phosphorylating respiration were calculated from the mitochondrial parameters shown in Fig. 4 and Fig. 5. Data represent mean ± SD, *n* = 3 (*P < 0.05, **P < 0.01, ***P < 0.001, ****P < 0.0001 versus control)

Respiratory flux control ratios can be derived from the mitochondrial functional parameters, namely State_apparent_, RCR, coupling efficiency and phosphorylating respiration (Fig. 6). (Figure 6 a) displayed the State_apparent_ for the five compounds in Psoraleae Fructus. As shown in (Fig. 6 a), the psoralidin State_apparent_ (3.47 ± 0.04) was significantly closer to State 3 compared to control (3.55 ± 0.04), bavachinin, bavachin and psoralen have the State_apparent_ at 3.76 ± 0.06, 3.67 ± 0.02, and 3.75 ± 0.04, respectively, closer to State 4 than control. The results (Fig. 6 b) showed that psoralidin RCR (2.42 ± 0.41) was significantly lower than control (8.01 ± 1.38) and the other four compounds. In Fig. 6 c, we observed that the coupling efficiency showed significant decrease in psoralidin (0.42 ± 0.08 vs. 0.76 ± 0.03 in control), while in Fig. 6d, phosphorylating respiration in psoralidin, bavachinin, bavachin and psoralen were lower with significant different than control (0.31 ± 0.05, 0.22 ± 0.05, 0.30 ± 0.02, 0.22 ± 0.01 vs. 0.39 ± 0.03 in control). Mitochondrial toxicants in XLGB resulted in reduced ATP turnover and impaired oxidative phosphorylation by inhibiting RCR, coupling efficiency and phosphorylation respiration, indicating that the cells in these compounds are unable to respond to energy demands.

**Fig. 7:**
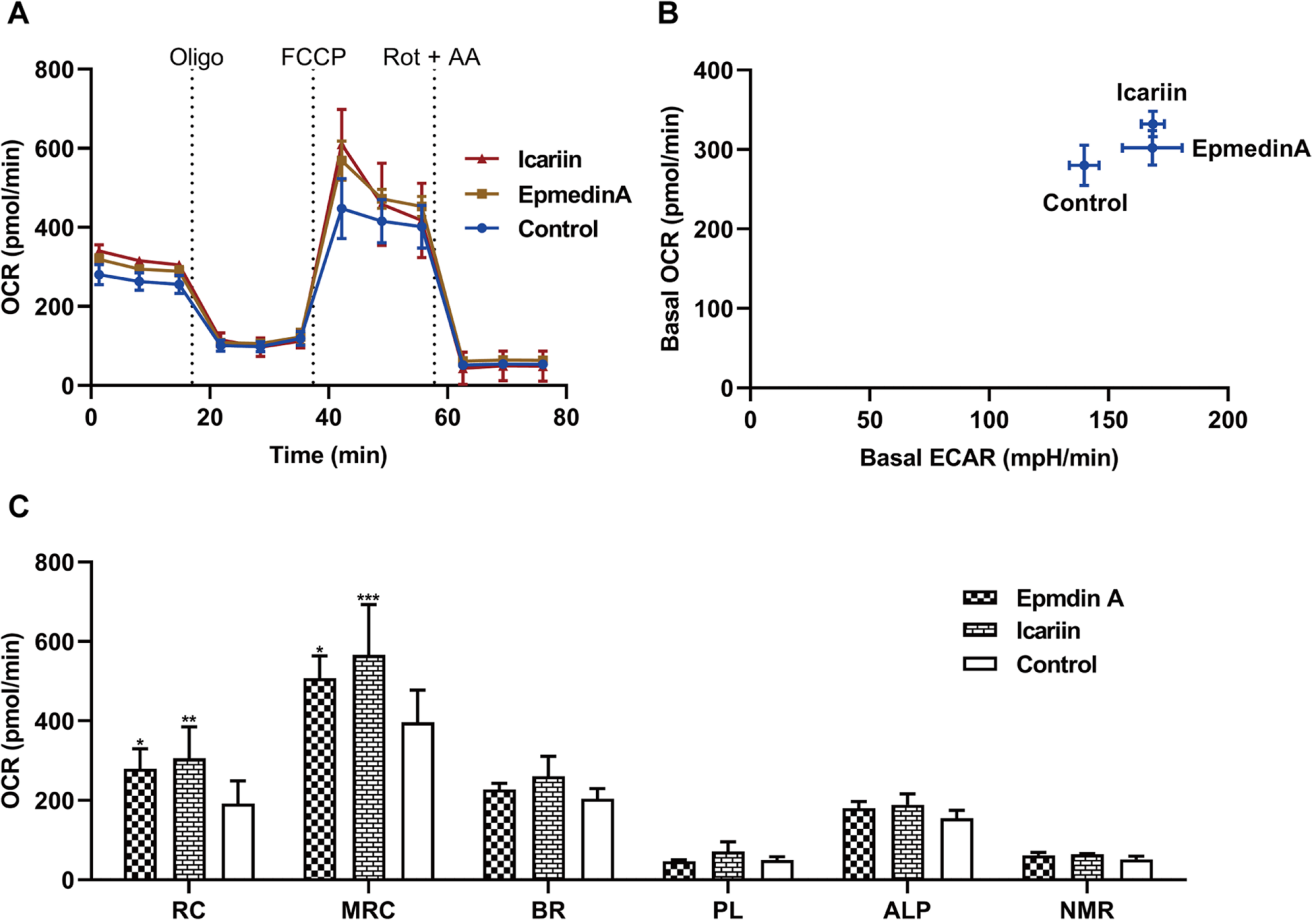
Measurement of bioenergetics profile of epmedin A and icariin. OCR (A) and ECAR (B) were measured in real time, the concentrations of epmedin A and icariin were at 238.44 and 295.57 μM respectively. (C) Each mitochondrial parameter was calculated derived from the data in A. Abbreviations and the corresponding parameters are as follows: reserve capacity (RC), maximal respiratory capacity (MRC), basal respiration (BR), proton leak (PL), ATP-linked production (ALP), and non-mitochondrial respiration (NMR). Data represent mean ± SD, *n* = 3 (*P < 0.05, **P < 0.01, ***P < 0.001 versus control OCR)

Although Epimedii Folium accounts for a relatively large proportion in XLGB, and the IC_50_ was lower than the other remaining herbal medicines except Psoraleae Fructus (91.77 vs. 73.22 μg/mL, in our previous work). Epimedii Folium showed no mitochondrial toxicity in Fig. 2, we also performed mitochondrial toxicity tests on the two main components in Epimedii Folium (epmedin A and icariin, with the contents of 4.37 and 3.86 μg/g respectively, in our previous work) using the Seahorse analyzer. As can be seen from Fig. 7, epmedin A and icariin showed the same response both at the increased basal respiration OCR and ECAR (Fig. 7 a-b), and the increased in maximal respiratory capacity and reserve capacity (Fig. 7 c). Further indicating that epmedin A and icariin are not the mitochondrial toxicants, and Epimedii Folium is not the herbal medicine that induced liver damage in XLGB.

### Network analysis of “Mitochondrial Toxicants – Targets” interaction in XLGB

**Fig. 8:**
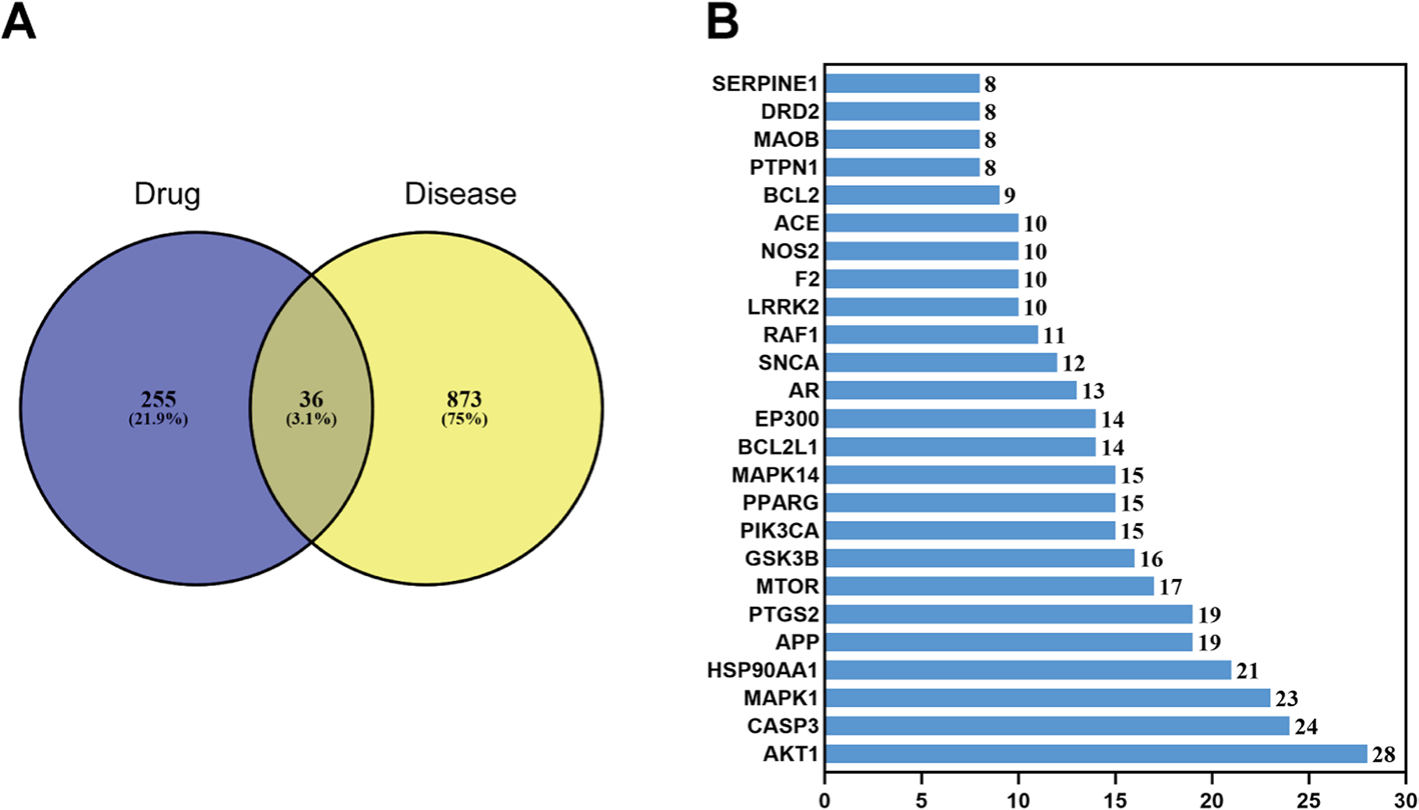
The PPI network for XLGB and targets analysis. (A) Venn diagram of XLGB targets and genes associated with mitochondrial dysfunction. (B) The top 25 genes in the PPI network are shown in the bar chart. The x-axis represents the number of target protein, and the target genes are represented on the y-axis

**Fig. 9:**
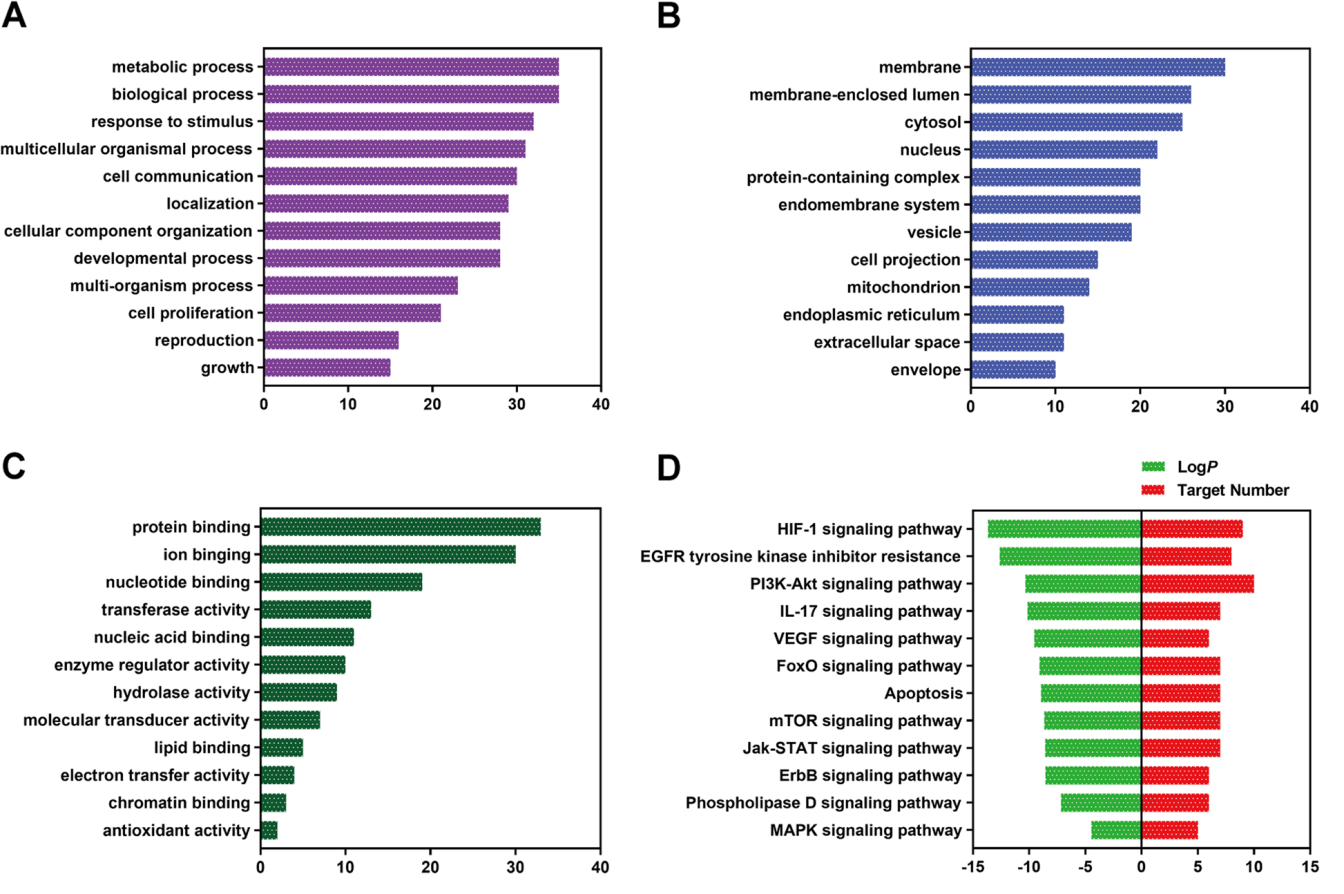
GO and KEGG analysis of potential targets of XLGB for Mitochondrial dysfunction. (A) Bar chart for biological process categories. (B) Bar chart for cellular component categories. (C) Bar chart for molecular function categories. (D) Enriched KEGG pathways of targets for Mitochondrial dysfunction

**Fig. 10:**
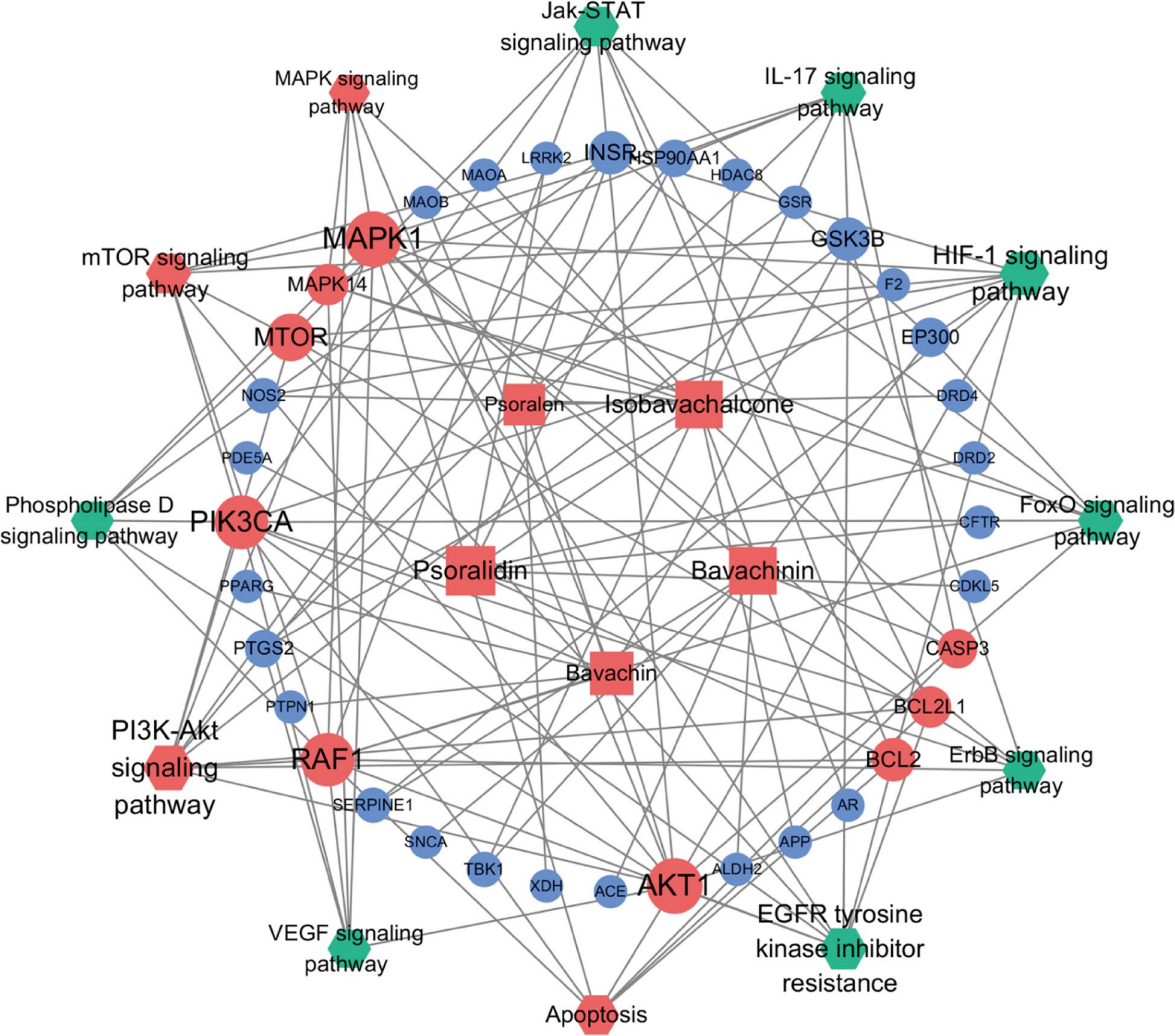
Compounds-compounds network of the top 12 signaling pathways. The square nodes represent compunds, the circles represent genes, and the hexagons represent signaling pathways

By searching Swiss Target Prediction and Comparative Toxicogenomics Database, a total of 255 targets related to the five mitochondrial toxicants were obtained. In addition, we searched GeneCards and Comparative Toxicogenomics Database with the keyword “Mitochondrial dysfunction” and identified a total of 873 genes. 255 XLGB targets and 873 mitochondrial dysfunction-related genes were compared through the Venny online tool, and 36 common targets were obtained (Fig. 8 A). The top 25 proteins in the network with multiple edges were plotted in Fig. 8 b. GO and KEGG enrichment analysis was performed using WebGestalt (Fig. 9). A complete network of compounds, targets and diseases was built using Cytoscape. As shown in Fig. 10, the degree value of a node indicates the number of routes connected in the network, and the bigger the shape, the higher the degree. The results showed that AKT1, PI3KCA, MAPK1, MTOR, RAF1, BCL2, BCL2L1, and CASP3 may be the most important targets. The influence of XLGB on mitochondrial dysfunction is closely related to PI3K-Akt signaling pathway (hsa 04151), mTOR signaling pathway (hsa 04150) and Apoptosis (hsa 04210).

### Effects of XLGB on protein expression

**Fig. 11:**
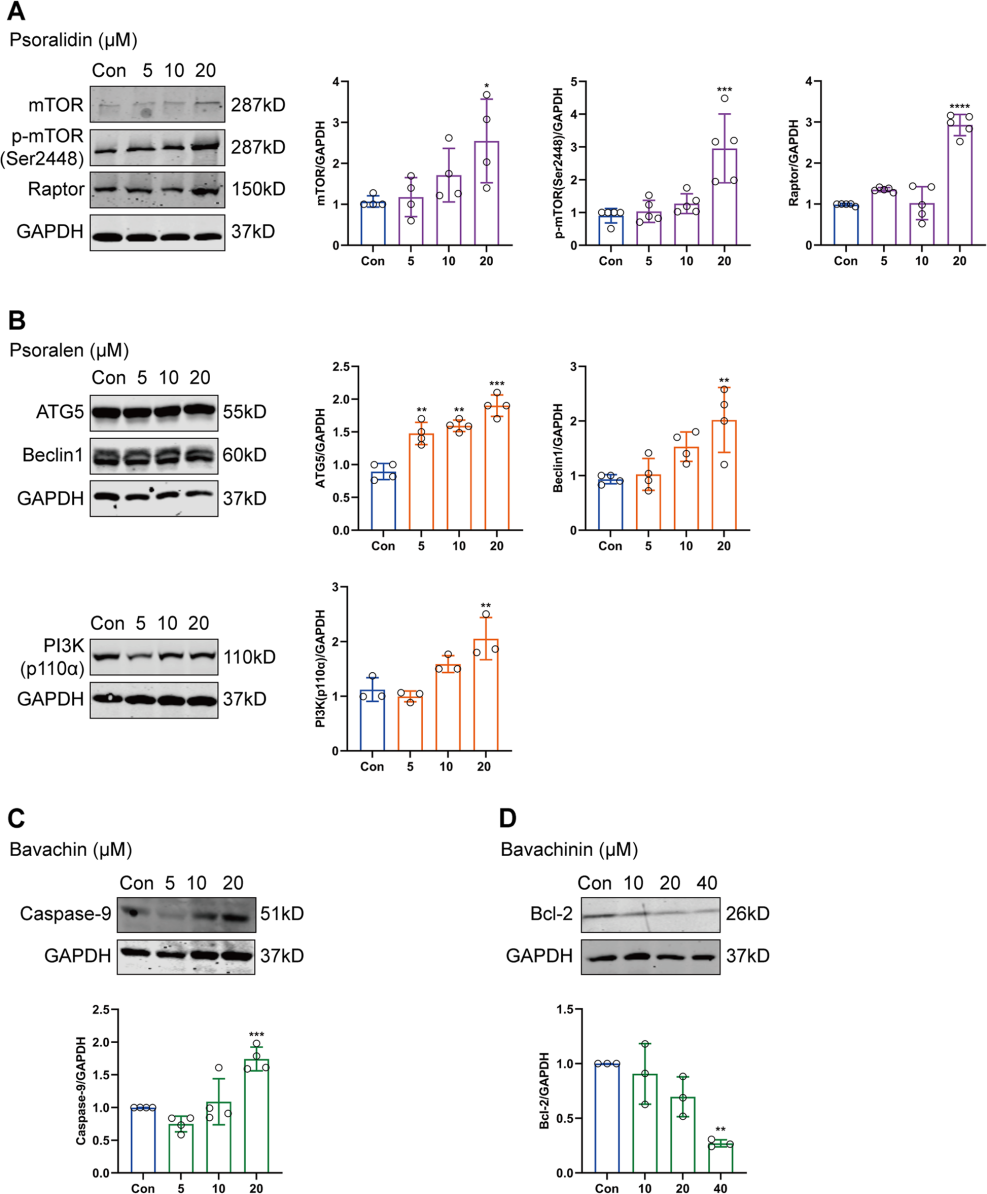
Western blot images and analyses of psoralidin, psoralen, bavachin and bavachinin. (A) Effects of psoralidin on mTOR, p-mTOR (Ser2448) and Raptor protein expression. (B) Effects of psoralen on ATG5, Beclin 1 and PI3K(p110α) protein expression. (C) Effects of bavachin on Caspase-9 protein expression. (D) Effects of bavachinin on Bcl-2 protein expression. (*P < 0.05, **P < 0.01, ***P < 0.001, ****P < 0.0001 versus control group)

On the basis of network pharmacology predictions, we investigated the molecular mechanisms of these five compounds. As shown in (Fig. 11 a-d), western blot exibited that the expressions of mTOR, p-mTOR (Ser2448), Raptor, PI3K (p110α), Beclin 1, ATG5 and Caspase-9 were up-regulated after treatment with psoralidin, psoralen and bavachin (Fig. 11 a-c), and after bavachinin treatment, the expressions of Bcl-2 was down-regulated (Fig. 11 d), suggesting that PI3K/mTOR signaling pathway and mitochondrial apoptosis may be involved in the liver damage caused by XLGB.

## Discussion

In this study, we determined the oxygen consumption rates of six herbal medicines in XLGB capsule and the effect of five major compounds in Psoraleae Fructus on the cellular bioenergetics, which was related to mitochondrial respiration in HepG2 cells. We found statistically significant decreases in maximal oxygen consumption of psoralidin, isobavachalcone and bavachinin, ATP-linked production of psoralidin, bavachinin and psoralen, indicating that the four compounds above can impair the function of mitochondria. It should be highlighted that in OCR, only basic respiration changes, such as bavachin, may be an early warning of mitochondrial dysfunction, which will subsequently affect reserve respiration and reduce ATP-linked respiration. In addition, reserve respiratory capacity is an important parameter that indicates the ability of cells to increase mitochondrial ATP synthesis capacity [21, 23]. When mitochondrial respiratory function is impaired, reserve capacity changes to keep ATP production capacity to maintain metabolic homeostasis. In the five compounds, psoralidin demonstrated a significant reduction in reserve capacity, indicating that the cells in psoralidin are less capable of maintaining reserve or spare respiratory capacity when bioenergetic demands increase.

State_apparent_ provides an evaluation of relative working state of mitochondrial under basal conditions and can be used as an indicator for cells to fall on after an intervention to infer the workload of mitochondrial [23]. Compared with the other four compounds, the State_apparent_ of psoralidin was closer to State 3, indicating an increased mitochondrial load of psoralidin. As a good indicator of mitochondrial dysfunction, RCR suggests the tight coupling between oxidative phosphorylation and respiration, and low RCR values generally indicate dysfunction [22]. The RCR of psoralidin was significantly lower than control and other four compounds, which means psoralidin destroyed mitochondrial function to a greater extent. Coupling efficiency and phosphorylating respiration give the fractions of basal mitochondrial oxygen consumption and maximal respiratory capacity used for ATP synthesis respectively. In our experiments, the value of coupling efficiency is significant lower in psoralidin, while in phosphorylating respiration, bavachinin, bavachin and psoralen are also lower with significant different than control, indicating that the mitochondrial respiration efficiency in these compounds for ATP production is reduced.

Mitochondrial toxicity is a common mechanism underlying DILI [3, 26, 27]. The ability of Seahorse assay to monitor the fluxes through bioenergetics pathways is of obvious value in assessing mitochondrial dysfunction. Many pharmaceutical companies have adopted this model for drug mitochondrial toxicity screening in preclinical research. Some studies have described in detail the relevance of cell type and culture medium on mitochondrial toxicity in Seahorse assay [28–30]. Although like most cancer cell lines, HepG2 cells have Warburg effect, which can alter the energy producing pathway from oxidative phosphorylation to the glycolytic pathway to sustain growth under anaerobic conditions. However, HepG2 cells, which can exhibit many of the genotypic and phenotypic characteristics of normal hepatocytes, are widely used for toxicity studies in the Seahorse assay [25]. Our protocol and data confirmed that Seahorse assay could be used to screen for mitochondrial dysfunction and performed well using HepG2 cells in glucose medium. In addition, mitochondrial toxicity has been observed at the concentrations below their half maximal inhibitory concentrations (except isobavachalcone, almost at IC_50_), suggesting that these compounds had already experienced mitochondrial dysfunction before cellular death.

Autophagy and apoptosis provide important roles in cell fate determination. The mitochondrion dependent (intrinsic) apoptosis pathway is an important mechanism in liver injury [31]. In most cases, autophagy represents an anti-apoptotic pro-survival process, while in some cases, autophagy helps to induce apoptosis, and eventually leads to cell death [32]. The cross-talk between autophagy and apoptosis has been proved by recent progress, which is regulated by genes such as Bcl-2, Beclin 1, Atg5 and caspases [33–35]. The best described relationship between autophagy proteins and apoptotic proteins is the complex relationship between Bcl-2 and Beclin 1 [36]. As an anti-apoptotic protein, Bcl-2 protein mainly acts on the mitochondrial membrane, sequestering pro-apoptotic proteins, preventing the permeability of mitochondrial membrane and the release of cytochrome c. Beclin 1, a key regulator of autophagy, cannot activate autophagy when bound to Bcl-2 protein. Caspase-mediated Beclin 1 cleavage can produce truncated proteins and cannot promote autophagy too. Instead, it promotes mitochondrial release of pro-apoptotic factors to promote apoptosis [37]. It is the same as another autophagy protein, Atg5. Atg5 is a protein necessary for autophagosome formation and regulation of autophagy, and it can also increase the sensitivity to apoptotic stimuli. Overexpression of ATG5 exhibited apoptotic features such as cell contraction and nuclear condensation [38]. Calpain-dependent Atg5 cleavage produced a truncated product translocated from the cytoplasm to the mitochondria to inhibit Bcl-2 protein and promote apoptosis [39]. mTOR, as a negative regulatory factor of autophagy, inhibits autophagy activation, and is regulated by the upstream signal of PI3K/Akt pathway [40]. In our experiments, we found that psoralidin and psoralen inhibited autophagy by up-regulating mTOR, p-mTOR, Raptor and PI3K. Bavachin and bavachinin induced mitochondrial apoptosis by up-regulating caspase-9 and down-regulating Bcl-2, respectively. Beclin 1 and ATG5 may play a cross-talk role in linking autophagy and apoptosis.

## Conclusion

In this paper, we demonstrate that the hepatotoxicity of XLGB is associated with mitochondrial damage. We identified Psoraleae Fructus as the target plant medicine responsible for the mitochondrial toxicity of XLGB. Investigation into cellular bioenergetics of the compounds in Psoraleae Fructus showed that psoralidin, isobavachalcone, bavachinin, bavachin and psoralen showed mitochondrial damage, especially psoralidin displayed significant reduction in reserve capacity and respiratory control ratios. The molecular mechanism of hepatotoxicity is related to the activation of PI3K/mTOR signaling pathway to inhibit autophagy and induce mitochondrial apoptosis.

## Supplementary Information

**Additional file 1.** Additional file

## Data Availability

All data generated or anaylzed during this study are available from the corresponding author.

## Abbreviations

XLGB: Xian-Ling-Gu-Bao capsule
TCM: traditional Chinese medicine
DILI: drug-induced liver injury
iDILI: idiosyncratic drug-induced liver injury
HILI: herbal-induced liver injury
FDA: Food and Drug Administration
CFDA: China Food and Drug Administration
OTC: over the counter
OVX rats: ovariectomized rats
SD rats: Sprague Dawley rats
XF: Extracellular Flux
OCR: oxygen consumption rate
ECAR: extracellular acidification rate
FCCP: carbonyl cyanide-p-(trifluoromethoxy) phenylhydrazone
RCR: respiration control ratio
ATP: adenosine triphosphate
HPLC: high performance liquid chromatography
HPLC-DAD: high performance liquid chromatography-diode array detection
HPLC-TOF-MS: high performance liquid chromatography-time of flight-mass spectrometry
ESI: electron spray ionization
DMEM: dulbecco’s modified eagle medium
PBS: phosphate buffer saline
SDS-PAGE: sodium dodecyl sulfate-polyacrylamide gel electrophoresis
BSA: albumin from bovine serum
TBST: Tris Buffered Saline with Tween
CCK-8: cell counting kit-8
PI3K: phosphatidylinositol 3-kinase
AKT: protein kinase B
mTOR: mammalian target of rapamycin
p-mTOR: phosphorylated mammalian target of rapamycin
Bcl-2: B-cell lymphoma-2
MAPK: mitogen-activated protein kinase.
