# Supplementary material for: Mitochondrial toxicants in Xian-Ling-Gu-Bao induce liver injury by regulating the PI3K/mTOR signaling pathway: an in vitro study
Source: BMC Complement Med Ther. 2022 Dec 1;22:317. doi: 10.1186/s12906-022-03798-5 (PMC9716976; doi:10.1186/s12906-022-03798-5)

Supplementary Information

Effect of five compounds in Psoraleae Fructus on cell viability. (Data represent mean ± SD, n = 3)

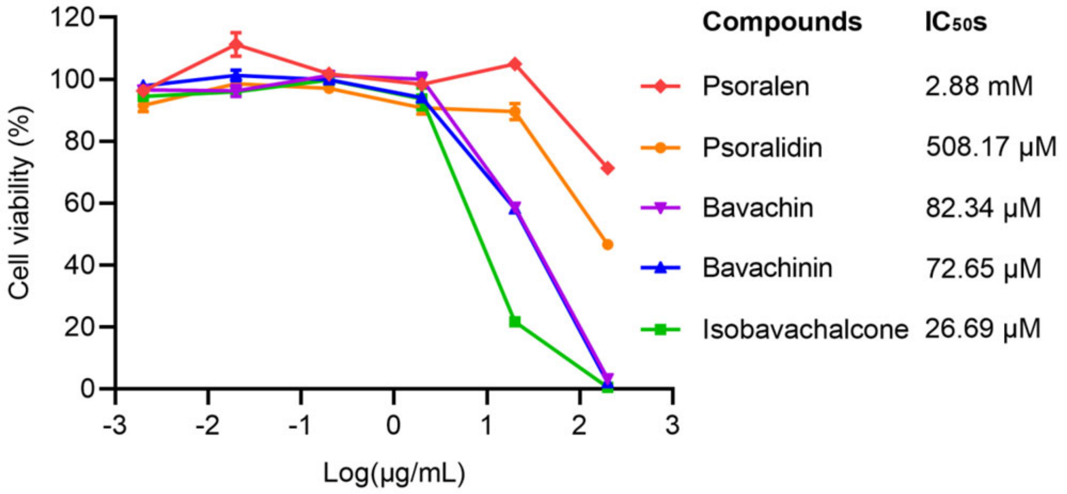

Figure 11A Western blot original strips of mTOR, p-mTOR(Ser2448) and Raptor.

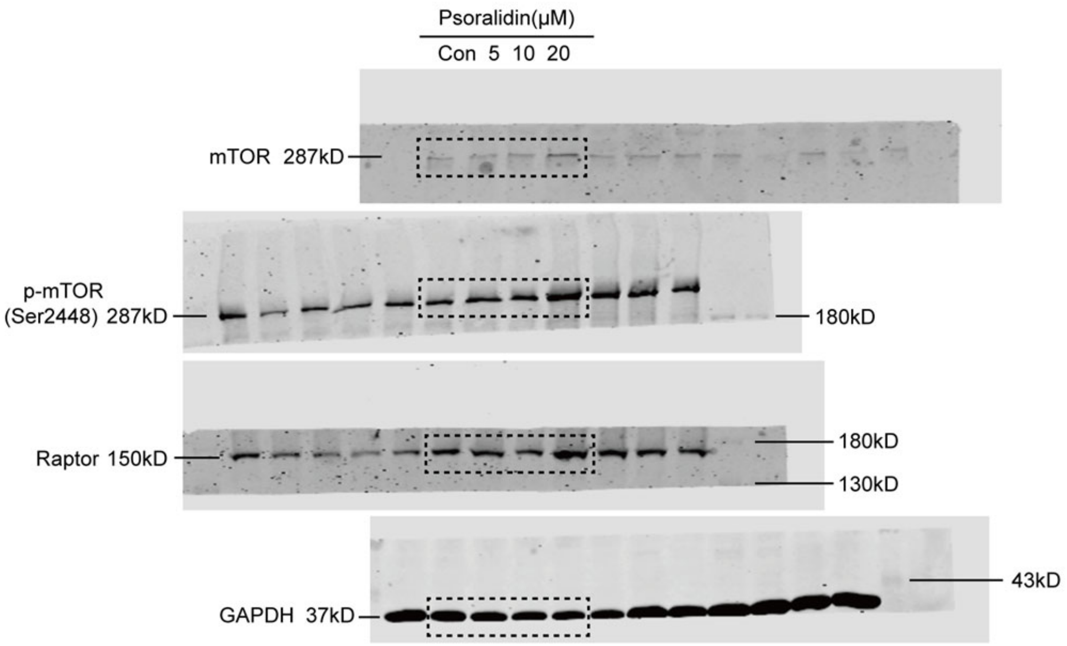

Figure 11B Western blot original strips of ATG5, Beclin1 and PI3K (p110α).

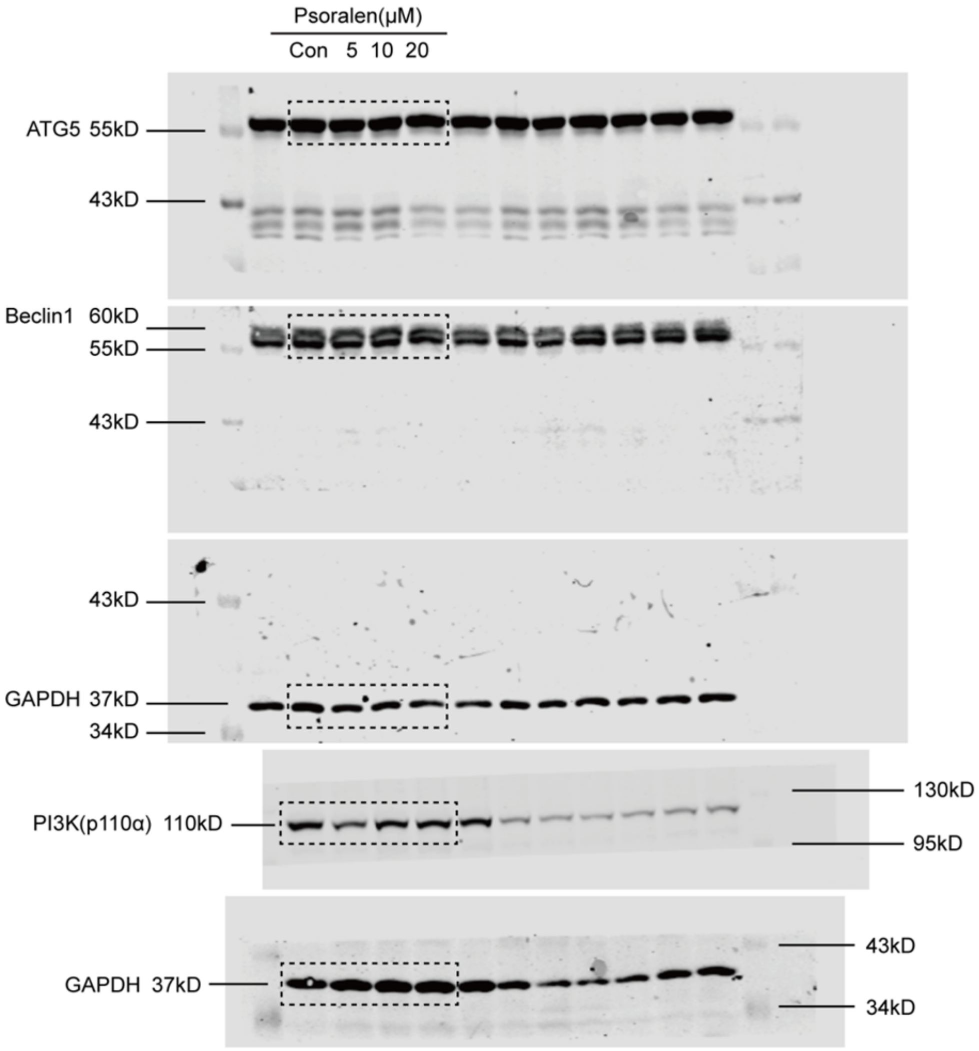

Figure 11C Western blot original strip of Caspase-9.

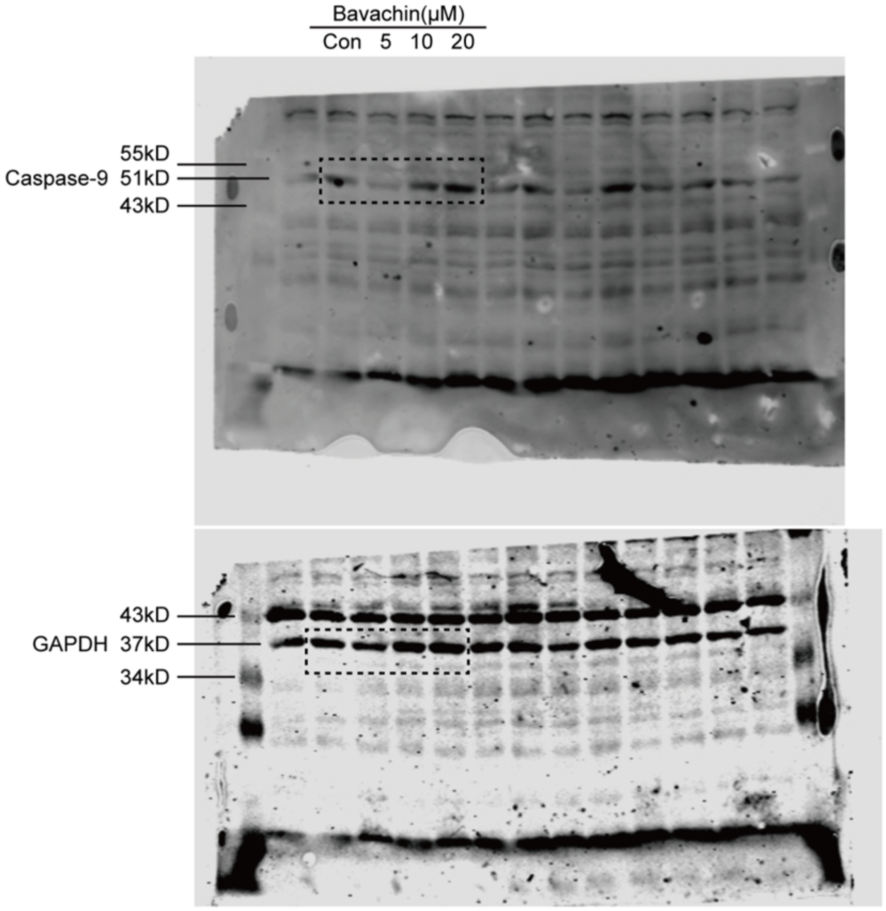

Figure 11D Western blot original strip of Bcl-2.

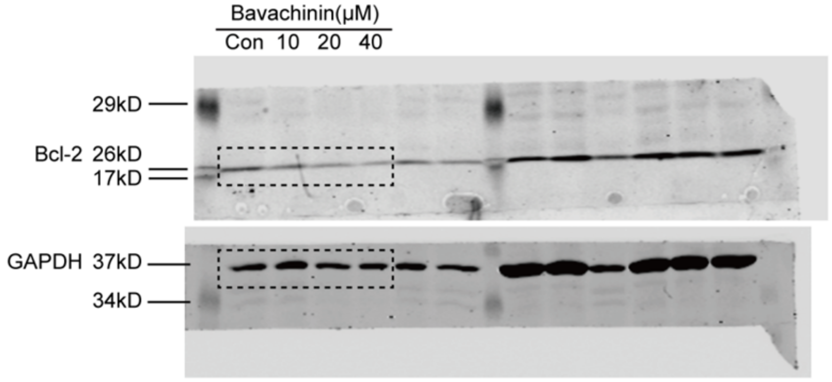

Supplement: Supplementary file 1 — Additional file 1. Additional file [file 12906_2022_3798_MOESM1_ESM.pdf]
